# [^11^C]Elacridar as a novel P-glycoprotein PET tracer, assessment of whole-body distribution and radiation dosimetry in humans

**DOI:** 10.1186/1471-2210-11-S2-A46

**Published:** 2011-09-05

**Authors:** Martin Bauer, Markus Zeitlinger, Georg Dobrozemsky, Cécile Philippe, Markus Müller, Oliver Langer

**Affiliations:** 1 Department of Clinical Pharmacology, Division of Clinical Pharmacokinetics and Imaging, Medical University of Vienna, 1090 Vienna, Austria; 2 Department of Nuclear Medicine, Medical University of Vienna, 1090 Vienna, Austria; 3 Health and Environment Department, Molecular Medicine, AIT Austrian Institute of Technology GmbH, 2444 Seibersdorf, Austria

## Background

The ATP-binding cassette transporter P-glycoprotein (P-gp) is expressed at the blood-brain barrier (BBB) where it protects the brain from toxic substances and xenobiotics by active efflux transport. The ^11^C-labelled third-generation P-gp inhibitor [^11^C]elacridar was developed as a positron emission tomography (PET) tracer for the *in vivo* quantification of P-gp expression levels in different organs. The aim of this study was to provide human dosimetry estimates for [^11^C]elacridar based on whole-body PET.

## Methods

Whole-body low dose computed tomography (CT) and dynamic and static whole-body PET scans were acquired in 4 healthy subjects for a total of 100 min after i.v. injection of 400 ± 8 MBq of [^11^C]elacridar using a Siemens Biograph scanner. Volumes of interest were placed in the brain, liver, pancreas, gallbladder, kidneys, lung, muscle, heart, spleen, bone marrow and bile by using ROVER (v. 2.0.31, ABX, Germany) software. Residence times were derived by spreadsheet calculation and adapted to the standard human model. Organ doses and effective dose were calculated utilizing the OLINDA (v. 1.1, Vanderbilt University) dosimetry program.

## Results

Organs with highest radiation burden included pancreas, spleen, liver and gallbladder wall. Furthermore, lungs, heart wall and kidneys received above average organ doses. As excretory organ the gallbladder was identified. Monoexponential fitting of activity overlying the gallbladder suggested that >95% of activity was excreted via the bile. The calculated effective dose was 7.0×10^−3^ mSv/MBq yielding 2.8 mSv for an injected amount of 400 MBq of [^11^C]elacridar.

## Conclusions

The estimated radiation burden of [^11^C]elacridar is in the range of other ^11^C-labeled PET tracers and would allow multiple PET examinations of the same subject per year.

